# Chemical inhibition of MrkH-dependent activation of type 3 fimbriae synthesis and biofilm formation by *Klebsiella pneumoniae*

**DOI:** 10.1038/s41522-025-00834-3

**Published:** 2025-11-20

**Authors:** Jonathan J. Wilksch, Jason W. H. Tan, Tracy L. Nero, Dianna M. Hocking, Vicki Bennett-Wood, Nancy Wang, Stefanie-Ann Zavras, Carl H. Schiesser, Marija Tauschek, Mark A. Schembri, Trevor Lithgow, Elizabeth L. Hartland, Roy M. Robins-Browne, Michael W. Parker, Ji Yang, Richard A. Strugnell

**Affiliations:** 1 Department of Microbiology and Immunology, The University of Melbourne, The Peter Doherty Institute for Infection and Immunity, Parkville, VIC Australia; 2 Infection & Immunity Program, Biomedicine Discovery Institute & Department of Microbiology, Monash University, Clayton, VIC Australia; 3 Department of Biochemistry and Pharmacology, Bio21 Molecular Science and Biotechnology Institute, The University of Melbourne, Parkville, VIC Australia; 4 ARC Centre of Excellence for Free Radical Chemistry and Biotechnology and the School of Chemistry and Bio21 Molecular Science and Biotechnology Institute, The University of Melbourne, Parkville, VIC Australia; 5 Institute for Molecular Bioscience (IMB), and School of Chemistry and Molecular Biosciences, University of Queensland, Brisbane, QLD Australia; 6 Structural Biology Laboratory, St Vincent’s Institute of Medical Research, Melbourne, VIC Australia; 7 Present Address: Murdoch Children’s Research Institute, Royal Children’s Hospital, Parkville, VIC Australia; 8 Present Address: Centre for Innate Immunity and Infectious Diseases, Hudson Institute of Medical Research, Australia and Department of Molecular and Translational Science, Monash University, Clayton, VIC Australia

**Keywords:** Biological techniques, Microbiology

## Abstract

Biofilm formation by *Klebsiella pneumoniae* is mediated by the type 3 fimbriae Mrk, and regulated by MrkH and 3’,5’-cyclic diguanylic acid (c-di-GMP). We sought to identify specific chemical inhibitors of *K. pneumoniae* biofilm formation that reduced the activity of MrkH. A compound N-(3-cyano-5,6,7,8-tetrahydro-4H-cyclohepta[b]thien-2-yl)-2-methoxybenzamide, JT71, reduced *K. pneumoniae mrkA* promoter activity and biofilm formation by 50% without affecting cell viability. Western blot analysis, hemagglutination assays, electron microscopy and qPCR showed that JT71 reduced type 3 fimbriae production, and transcription of *mrkA* and *mrkH*. JT71 demonstrated activity against other clinical and multi-drug resistant *K. pneumoniae* isolates, and a type 3 fimbriate-positive *Citrobacter koseri* strain. In silico molecule docking was used to illustrate that JT71 could bind directly to the MrkH protein and block its activity. JT71 possesses promising drug-likeness properties and is non-toxic to mammalian cells. Chemical inhibition of transcriptional regulators that control fimbriae expression can inhibit bacterial biofilm formation.

## Introduction

The *Klebsiella pneumoniae* species complex (KpSC) is a heterogeneous group of Gram-negative, opportunistic bacteria that are responsible for community and nosocomial infections, especially amongst immunocompromised patients^1–3^. An important mechanism of pathogenesis in hospital environments involves the formation of biofilms, which are broadly defined as surface-associated communities of bacterial cells. The presence of biofilms on medical devices used in health-care settings, such as catheters and implants, is frequently associated with infections, and places a significant burden on health care systems^4–6^.

The widespread and sometimes indiscriminate usage of conventional antimicrobials to treat bacterial infections has inevitably exerted a strong selective pressure on KpSC, resulting in the emergence of multidrug-resistant (MDR) strains, such as those carrying genes encoding for extended-spectrum β-lactamases^7–9^. A novel carbapenem-hydrolysing β-lactamase, KPC-1 from *K. pneumoniae*, was first reported in 2001 in the USA and subsequently worldwide^10^, and confers resistance to nearly all β-lactams. The continued spread of these MDR pathogens represents a major threat to global human health^11–15^, and genomics of a pan-resistant *K. pneumoniae* strain revealed the critical role of mobile resistance elements in driving new resistance^16^. It is evident that biofilm-associated cells exhibit significantly enhanced phenotypic resistance to antimicrobial agents and disinfectants^17,18^; in some cases, the concentrations of antibiotics needed to reach bactericidal activity against bacteria with biofilms can be 10–1000 times higher than for planktonic cells, making them difficult to eradicate^19,20^. The close proximity of bacteria to each other within biofilms facilitates horizontal gene transfer, including resistance genes^21^.

An emerging strategy to combat bacterial pathogens that have become recalcitrant to conventional antibiotic treatment is to specifically target microbial virulence factors without affecting cell viability^22^. For example, drugs that inhibit virulence gene expression might lead to a reduction in a bacterium’s ability to colonise in vitro or in vivo surfaces, or evade a host’s immune system, without imposing a selective pressure for the development of resistance that occurs when bacterial growth is targeted^23,24^.

Most efforts in identifying novel virulence inhibitors place strong emphasis on targets such as toxin function and delivery^25–27^, regulators of virulence expression^28,29^, or the assembly of fimbriae^30^. None of these drugs, which address virulence, has been approved for use in humans. Anti-biofilm drugs targeting *K. pneumoniae*, such as diguanylate cyclase enzyme inhibitors^31^ or an iron antagonising molecule^32^ have been reported. These approaches are, like most current antibiotics, not specific for pathogens and risk changing the ‘protective’ microbiome.

Previous studies have shown that *K. pneumoniae* biofilm formation on a range of surfaces is mediated by the type 3 fimbriae, encoded by the *mrkABCDF* operon^33–35^. MrkA is the major subunit of the fimbriae that facilitates binding to abiotic surfaces, while MrkB and MrkC function as the periplasmic chaperone and the outer membrane usher, respectively^34,36^. Through the MrkD adhesin tip of the fimbriae, *K. pneumoniae* exhibits adherence to extracellular matrix proteins such as type IV and/or type V collagen, human bronchial cells, and basement membrane regions of epithelial cells^37,38^. The *mrkABCDF* operon, which is transcribed by a single promoter located upstream of *mrkA*, is positively regulated by a 3’,5’-cyclic diguanylic acid (c-di-GMP)-dependent transcriptional activator called MrkH, a PilZ domain protein^39–41^. When *mrkH* is absent or mutated, the *mrkABCDF* operon is transcriptionally inactive^39–41^ and biofilm formation is heavily mitigated^39^. However, when present and in association with c-di-GMP, MrkH binds to a nucleotide sequence known as the “MrkH box” located upstream of the type 3 fimbrial operon, to facilitate recruitment of RNA polymerase to the *mrkA* promoter for transcription of the *mrkABCDF* operon^39,42^. We have also reported that MrkH functions as an auto-activator for the *mrkHI* genes in a mechanism analogous to its function at the *mrkA* promoter^43^. The *mrkI* gene, which encodes another transcriptional regulator involved in type 3 fimbriae expression, is co-transcribed with *mrkH*^39–41^.

Given the critical importance of MrkH in activating *K. pneumoniae* biofilm formation, we sought to identify inhibitors of MrkH that could lead to the development of new anti-biofilm approaches (e.g., incorporation of chemicals into medical plastics to prevent bacterial attachment and subsequent biofilm formation). In this study, we screened a small compound library and identified one compound, which we named JT71, that specifically inhibited MrkH-dependent *mrk* transcription, type 3 fimbriae synthesis, and biofilm formation by *K. pneumoniae*.

## Results

### Design of a phenotypic screen and identification of a MrkH inhibitor

We developed a phenotypic screen of a synthetic compound library (ChemBridge ‘Microformats’) to identify compounds that specifically inhibit MrkH function (i.e., by inhibiting MrkH-mediated transcription of the *mrkA* promoter). The inhibitor screen used *Escherichia coli* strain MC4100 harbouring a MrkH-expressing plasmid (pACYC184-*mrkH*) and the single-copy plasmid pMU*mrkA*-*lacZ*, which carries a transcriptional fusion of the *mrkA* promoter (inducible by c-di-GMP via MrkH) upstream of the *lacZ* gene that encodes β-galactosidase^39^. Previous studies have shown that *E. coli* MC4100 carrying pMU*mrkA*-*lacZ* expresses very low β-galactosidase levels^39,42^. However, in the presence of pACYC184-*mrkH*, the β-galactosidase activity increases by over 300-fold^39,42^. Using this reporter system to measure MrkH-mediated activation of transcription from the *mrkA* promoter offered a simple method to screen for chemical inhibitors of MrkH. To eliminate false positives in the screen, a control *E. coli* MC4100 strain containing an unrelated transcriptional regulatory system (pACYC184-*aggR/aap*-*lacZ*)^44,45^ was run in parallel with the test strains. AggR regulates virulence genes in enteroaggregative *E. coli* and does not control type 3 fimbriae, making it a suitable negative control in this assay. Compounds that inhibited *E. coli* growth or β-galactosidase production in both test and control strains were not examined further.

**Fig. 1:**
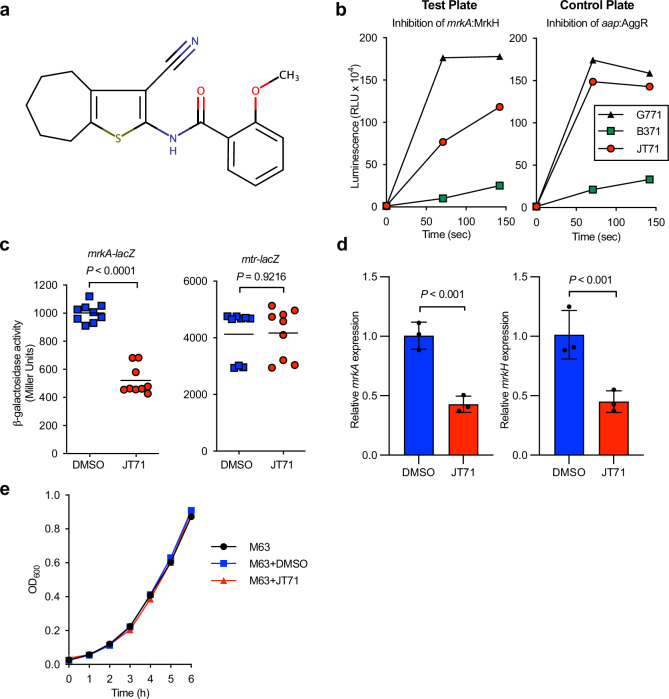
Effects of JT71 on MrkH-mediated transcription and bacterial growth. **a** Chemical structure of JT71. **b** Luminescence-based β-galactosidase screen of *E. coli* MC4100 containing either pMU*mrkA*-*lacZ* and pACYC184-*mrkH* (test plate) or pMU*aap*-*lacZ* and pACYC184-*aggR* (control plate), following treatment with ChemBridge library compounds. The activity of the hit compound JT71 was assessed relative to G771, a representative non-inhibitory compound on the same plate, while B371 represents a false positive that inhibited both reporters. Data shown are single measurements from the primary screen. **c** β-galactosidase activity from the *mrkA* promoter reporter in *K. pneumoniae* Δ*lacZ* in the presence of 50 µM JT71 or 1% DMSO. Activity from the *mtr*-*lacZ* control promoter is shown in parallel. Symbols represent biological replicates pooled from three independent assays; horizontal lines indicate the mean. Statistical analysis was performed using an unpaired two-tailed *t* test. **d** qRT-PCR analysis of *K. pneumoniae* AJ218 grown in the presence of 50 µM JT71 or 1% DMSO. Transcript levels of *mrkA* and *mrkH* were quantified and normalised to *rpoD*. Expression values are presented as fold change relative to the DMSO control. Horizontal bars indicate the mean ± SD of three biological replicates. Statistical analysis was performed using an unpaired two-tailed *t* test. **e** Static growth of *K. pneumoniae* AJ218 (OD_600_) over 6 hours in M63 media containing 50 µM JT71 or 1% DMSO. Data are shown as the mean ± SD from three independent assays.

Screening of 13,440 synthetic compounds in the *E. coli* host strain identified the compound *N*-(3-cyano-5,6,7,8-tetrahydro-4H-cyclohepta[b]thien-2-yl)-2-methoxybenzamide (Fig. 1a; JT71) as a potent inhibitor of MrkH activity. Relative to the non-inhibitory reference compound G771, JT71 reduced *mrkA*-*lacZ* reporter output by approximately 56% at 71 s and 34% at 142 s when tested at 100 µM, while its effect on the *aap*-*lacZ* reporter was comparatively minor (15% and 10% reductions, respectively; Fig. 1b). Also shown in Fig. 1b is compound B371, which inhibited both reporters and was classified as a false positive due to non-specific or toxic effects. JT71 selectively inhibited luminescence only in the *mrkA-lacZ* reporter strain, suggesting target specificity. The chemical structures of B371 and G771 are provided in Supplementary Fig. S1.

To validate this finding in the native host, JT71 was tested in two strains of *K. pneumoniae* AJ218 (Δ*lacZ*) carrying either pMU*mrkA*-*lacZ* (test strain) or pACYC177-*tyrR*/pMU*mtr*-*lacZ* (control strain)^29^. At 50 µM, JT71 inhibited MrkH-mediated *mrkA* promoter activity by approximately 50%, while having no measurable effect on TyrR-dependent *mtr* expression (Fig. 1c). The use of two unrelated regulatory systems (*aggR*/aap and *tryR*/*mtr*) across both stages of the assay helped confirm the specificity of JT71 and rule out off-target effects on general transcription or β-galactosidase activity. To determine whether the observed effects were due to general toxicity, *K. pneumoniae* was cultured in M63 minimal media with or without 50 µM JT71. M63 media was used in place of LB for downstream assays, as it promotes more consistent expression of type 3 fimbriae and provides a defined environment for assessing compound activity.

To confirm that JT71 inhibits MrkH-dependent transcription of the *mrkABCDF* and *mrkHI* operons, we quantified *mrkA* and *mrkH* mRNA levels in *K. pneumoniae* AJ218 grown with or without 50 µM JT71 using quantitative real-time PCR (qRT-PCR). Treatment with JT71 led to a 2.4-fold reduction in *mrkA* transcript levels compared to the DMSO control (*P* = 0.0016), and a 2.4-fold reduction in *mrkH* transcript levels (*P* = 0.0070) (Fig. 1d). These results demonstrate that JT71 inhibits transcription from both the *mrkA* and *mrkH* promoters, consistent with its proposed role as an inhibitor of MrkH function. No differences in growth were observed over a 6-hour incubation, confirming that JT71 did not impair bacterial viability under these conditions (Fig. 1e).

### JT71 reduces type 3 fimbriae production of *K. pneumoniae*

**Fig. 2:**
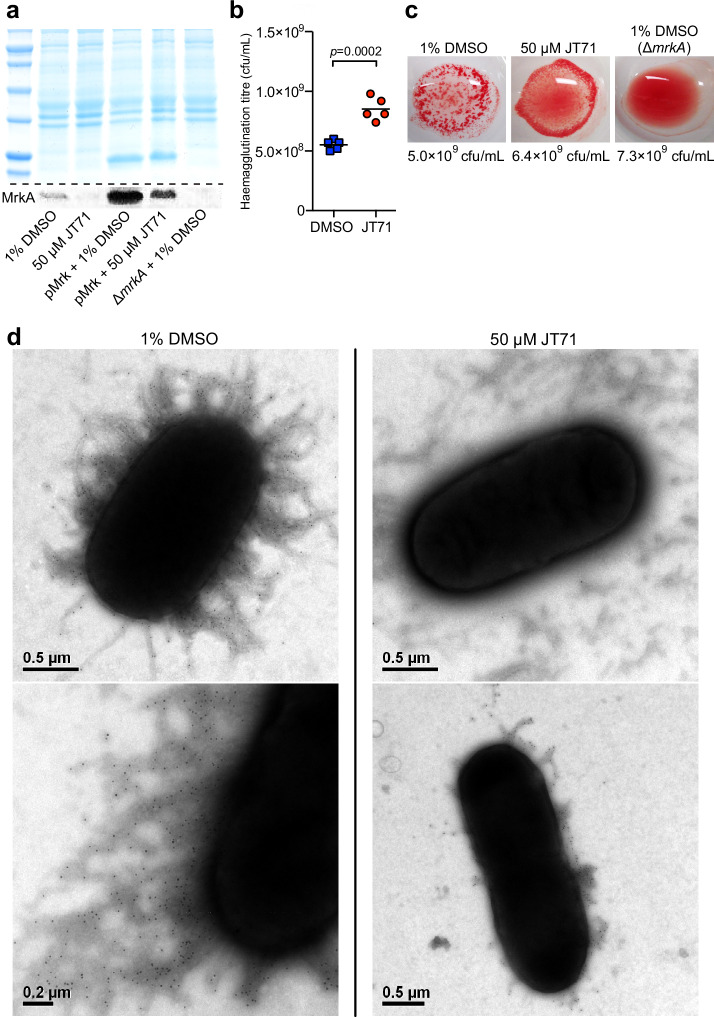
Analysis of type 3 fimbriae production in *K. pneumoniae* in the presence of JT71. **a** SDS-PAGE of whole-cell lysates and Western blot of MrkA from *K. pneumoniae* grown with or without 50 µM JT71. *K. pneumoniae* containing pMrk (*mrkABCDF*) was used to overexpress type 3 fimbriae. A type 3 fimbriae mutant (Δ*mrkA*) was used as a negative control. **b** Hemagglutination assay showing the lowest bacterial concentration (cfu/mL) required to agglutinate tanned sheep erythrocytes. Horizontal bars represent the mean, and data points shown are biological replicates from one representative experiment of two independent experiments performed. Statistical analysis was performed using an unpaired two-tailed *t* test. **c** Representative images of hemagglutination reactions with *K. pneumoniae* at the indicated bacterial concentrations grown with or without 50 µM JT71. **d** Representative TEM images of *K. pneumoniae* immunogold-labelled with anti-MrkA antibodies. Cells were grown with or without 50 µM JT71. Immunogold particles (black dots) indicate surface-localised MrkA. Scale bars are shown.

To confirm that JT71 inhibits MrkH function and determine whether this results in reduced type 3 fimbriae expression, we performed assays to detect MrkA production. Whole-cell lysates from *K. pneumoniae* were separated by SDS-PAGE and analysed by Western blot using anti-MrkA antisera to detect the major fimbrial subunit. The upper panel of Fig. 2a shows a Coomassie-stained gel used to verify equal protein loading across lanes. The lower panel shows the corresponding Western blot, comparing untreated (1% DMSO) *K. pneumoniae* samples (wild-type and Δ*mrkA* complemented with pACYC184-*mrkABCDF*) with samples treated with 50 µM JT71. MrkA production was markedly reduced in JT71-treated samples relative to untreated controls. The complemented strain, which overexpresses *mrkABCDF*, exhibited a more intense MrkA signal, as expected, and was used to enhance detection sensitivity due to low MrkA expression in the wild-type background.

The type 3 fimbriae of *K. pneumoniae* can be selectively detected in a functional assay using tanned erythrocytes in a mannose-resistant hemagglutination reaction that involves the fimbrial adhesin tip protein MrkD^46^. We sought to determine whether JT71 reduces the production of type 3 fimbriae using this assay. Following growth of *K. pneumoniae* AJ218 for 6 hours statically in the presence of 50 µM JT71 or 1% DMSO, serial dilutions of bacteria were mixed with tanned sheep erythrocytes and allowed to agglutinate. The minimum bacterial density of *K. pneumoniae* required to agglutinate the erythrocytes was then determined. As expected, the negative control (*K. pneumoniae* Δ*mrkA*) failed to agglutinate erythrocytes even at the highest bacterial cell density tested (7.3 × 10^9^ cfu/mL). The average minimum bacterial density required to cause an agglutination reaction in *K. pneumoniae* wild-type was 5.5 × 10^8^ cfu/mL for the untreated sample (1% DMSO) and 8.5 × 10^8^ cfu/mL for the JT71-treated sample (50 µM) (Fig. 2b). Thus, JT71 significantly increased the number of *K. pneumoniae* cells required to agglutinate erythrocytes in a type 3 fimbriae-dependent manner (see Fig. 2c for representative images of agglutination reactions).

Transmission electron microscopy (TEM) with immunogold labelling was used to visually confirm whether JT71-treatment of *K. pneumoniae* caused reduced production of type 3 fimbriae on the cell surface. The cell surface of untreated bacteria (1% DMSO) was covered in large quantities of fimbriae up to 1 µm long that were bound to gold-labelled monoclonal MrkA antibodies (Fig. 2d). In contrast, JT71-treated bacteria showed a reduction in the number and length of labelled fimbriae on the cell surface.

Taken together, these observations demonstrated that JT71 is a type 3 fimbriae inhibitor that acts to significantly reduce the amount of MrkA subunit produced, leading to a reduction of type 3 fimbriae presented on the *K. pneumoniae* cell surface.

### Incubation of *K. pneumoniae* with JT71 inhibits biofilm formation

We have previously reported that MrkH promotes type 3 fimbriae production and biofilm formation on a number of medically relevant materials, including polystyrene and collagen-coated surfaces^29,39,43^. Having established that JT71 reduced MrkH-mediated transcription from the *mrkA* and *mrkH* promoters, causing a significant reduction in type 3 fimbriae production, we next examined whether this translated into reduced biofilm formation.

**Fig. 3:**
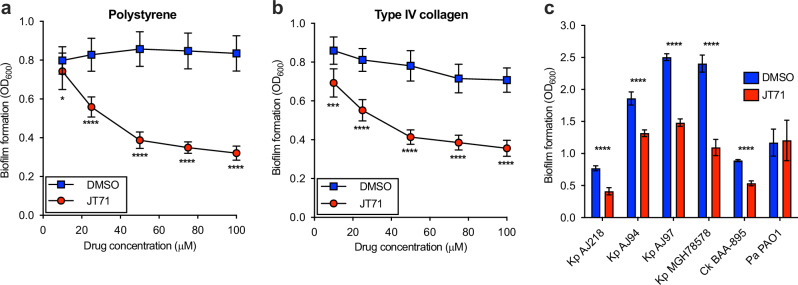
Inhibition of biofilm formation by JT71. Biofilm formation of *K. pneumoniae* AJ218 on uncoated polystyrene (**a**) and type IV collagen-coated polystyrene (**b**) grown in the presence of varying concentrations of JT71 or equivalent concentrations of DMSO solvent used. Biological replicates (*n* = 25–30) are pooled from three independent assays, and the mean ± 95% CI for each group is shown. **c** Biofilm formation of *K. pneumoniae* (Kp), *C. koseri* (Ck), and *P. aeruginosa* (Pa) strains grown with or without 50 µM JT71. Data are the mean ± 95% CI of biological replicates pooled from three independent assays. Each JT71-treated group was compared to controls that were treated with equivalent amounts of DMSO solvent using two-way ANOVA with the Bonferroni post-test, *P < 0.05; ***P < 0.001; ****P < 0.0001.

Biofilm assays were performed using *K. pneumoniae* AJ218 cultured in M63 minimal media on uncoated polystyrene and type IV collagen-coated polystyrene surfaces. Type IV collagen was selected to simulate host extracellular matrix conditions encountered on damaged tissue or indwelling medical devices, where type 3 fimbriae play a critical role in adherence^33,47^. JT71 significantly inhibited biofilm formation on both surfaces in a dose-dependent manner (Fig. 3). Maximum inhibition was observed at 100 µM, with a 60% reduction on uncoated polystyrene (Fig. 3a), and a 50% reduction on type IV collagen-coated surfaces (Fig. 3b). These findings confirm that blocking MrkH function with JT71 impairs the ability of *K. pneumoniae* to form biofilm under both abiotic and host-mimicking conditions.

To assess whether JT71 can reduce pre-formed biofilms, *K. pneumoniae* AJ218 biofilms were established for either 8 or 24 hours prior to treatment with 50 µM JT71 or 1% DMSO. Assays were performed either by adding the treatment directly to the existing medium or by replacing the medium with fresh treatment-containing medium. Biofilm biomass was then quantified over periods of up to 48 hours post-treatment (Supplementary Fig. S2). Across all conditions, JT71 did not significantly reduce biofilm biomass relative to DMSO controls (Wilcoxon signed-ranked test, *p* > 0.05). These findings indicate that JT71 lacks biofilm-eradicating activity but remains a potent inhibitor of biofilm formation during the early stages of development.

### JT71 inhibits biofilm formation in other type 3 fimbriated *K. pneumoniae* and *Citrobacter koseri* isolates

The chromosomal region encoding the *mrkABCDF* and *mrkHI* operons is highly conserved among *K. pneumoniae* strains, with nucleotide similarity exceeding 90% across sequenced genomes^39^. To determine whether JT71 has broader anti-biofilm activity within this species, we tested three additional clinical *K. pneumoniae* isolates (AJ94, AJ97, and MGH78578), all of which carry conserved *mrk* operons. Each strain showed a significant reduction in biofilm formation when cultured with 50 µM JT71 compared to DMSO-treated controls (Fig. 3c), consistent with inhibition of MrkH-dependent fimbriae production.

We next examined whether JT71 could inhibit biofilm formation in *C. koseri*, another type 3 fimbriae-producing species within the Enterobacteriaceae. *C. koseri* is a known cause of urinary tract infections, sepsis, and meningitis, particularly in neonates and immunocompromised adults^48–50^. It also carries a conserved *mrk* locus^51^, but robust biofilm formation under our assay conditions required elevated intracellular c-di-GMP levels. To promote biofilm formation and activate MrkH, we overexpressed the *yfiRNB* operon (encoding the diguanylate cyclase YfiN^39^) in wild-type *C. koseri* BAA-895 using pACYC184-*yfiRNB*. Treatment with 50 µM JT71 significantly reduced biofilm formation relative to DMSO controls (Fig. 3c), suggesting that the compound inhibits MrkH-dependent transcription in *C. koseri* as well.

To test whether JT71 affects biofilm formation in organisms lacking *mrk* genes, we examined the *Pseudomonas aeruginosa* model strain PAO1, which does not encode a MrkH homologue and type 3 fimbriae. JT71 had no measurable effect on PAO1 biofilm formation under the same conditions (Fig. 3c). These findings support JT71’s activity as being specific to type 3 fimbriae-producing species and consistent with its proposed mechanism of targeting MrkH-dependent regulatory pathways.

### Analysis of JT71 analogues

**Fig. 4:**
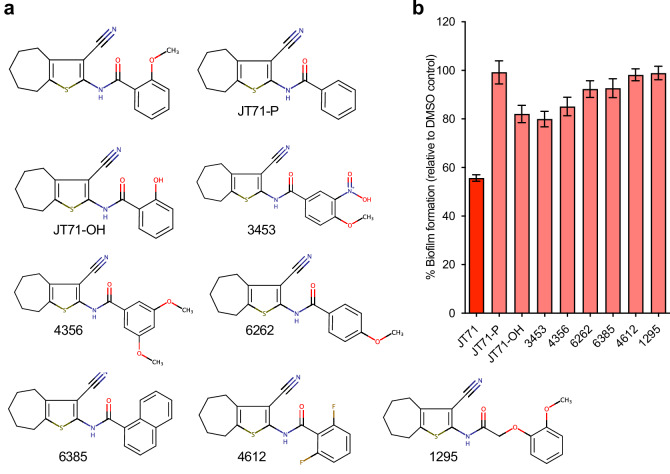
Structural analogues of JT71 and their effect on biofilm formation. **a** Chemical structures of JT71 and eight analogues selected to examine the importance of the ortho-methoxy substituent of the phenyl ring. **b** Biofilm formation by *K. pneumoniae* AJ218 in the presence of 50 µM JT71 or each analogue. Data represent the mean ± 95% CI of three independent assays.

Since JT71 emerged as a key hit compound, we sought to determine whether structural modifications could improve its biofilm-inhibitory activity. We focused on a small set of synthetically tractable modifications of the benzamide phenyl ring, while maintaining the 3-cyano-cycloheptathienyl part of the molecule, so that we could clearly observe the effect of the modifications on biofilm formation. The eight analogues were tested, along with JT71, at 50 µM in a *K. pneumoniae* AJ218 biofilm assay, with biofilm levels normalised to matched DMSO-treated controls. The ortho-methoxy group (i.e., O-CH3) of JT71 is a potential hydrogen bond acceptor (via the oxygen atom), and it also influences the orientation of the phenyl ring with respect to the amide moiety. Removal of the ortho-methoxy to produce JT71-P greatly reduces the ability of the compound to prevent biofilm formation (Fig. 4b), suggesting that an ortho-substituent is required for activity. Replacing the methoxy group with hydroxyl (i.e., compound JT71-OH) introduces the ability to both donate and accept hydrogen bonds in the ortho-position but decreases the size of the substituent while increasing its polarity (compared to the ortho-methoxy substituent). JT71-OH also has reduced anti-biofilm activity compared to the parent compound, JT71 (Fig. 4b). Fluorine is much smaller in size than a methoxy or hydroxyl group, and the introduction of a fluorine into the two ortho-positions (i.e., compound 4612) does not retain or improve activity. Incorporating the ortho and meta-positions of the phenyl ring into a napthyl ring system (i.e., compound 6385) also results in a loss of activity when compared to JT71 (Fig. 4b). Similarly, moving the methoxy substituent around the phenyl ring to the meta- or para-positions does not improve or retain activity (i.e., compounds 4356, 6262, and 3453). Increasing the distance between the amide moiety and the phenyl ring by inserting a methoxy linker (i.e., -OCH_2_-), to produce compound 1295, also results in a loss of anti-biofilm activity (Fig. 4b). In summary, all eight analogues showed markedly reduced anti-biofilm activity relative to JT71, demonstrating the importance of the ortho-methoxy substituent of the benzamide moiety of the parent compound.

### JT71 exhibits “drug-likeness” properties

The molecular weight of JT71 is 326.4 g/mol, which is less than the upper limit of 500 g/mol predicted to be optimal for orally active small molecule compounds. Likewise, the compound contains one hydrogen bond donor and four hydrogen bond acceptors, which are within the optimal range for these parameters^52^. Finally, JT71 exhibits a low polar surface area (tPSA = 90.4 Å^2^) and has a partition coefficient (clogP) of 4.13. Altogether, JT71 fully satisfies Lipinski’s rule of five (i.e., molecular weight < 500 g/mol, clogP <5, <5 hydrogen bond donors and <10 hydrogen bond acceptors^52,53^) and possesses drug-likeness properties^54^. Importantly, treatment of 50 µM JT71 was found to be non-toxic to mammalian HeLa cells when compared to untreated cells, as measured by the release of LDH, an indicator of cell death (Supplementary Fig. S3).

### Modelling the JT71-MrkH complex suggests two distinct inhibitory mechanisms

**Fig. 5:**
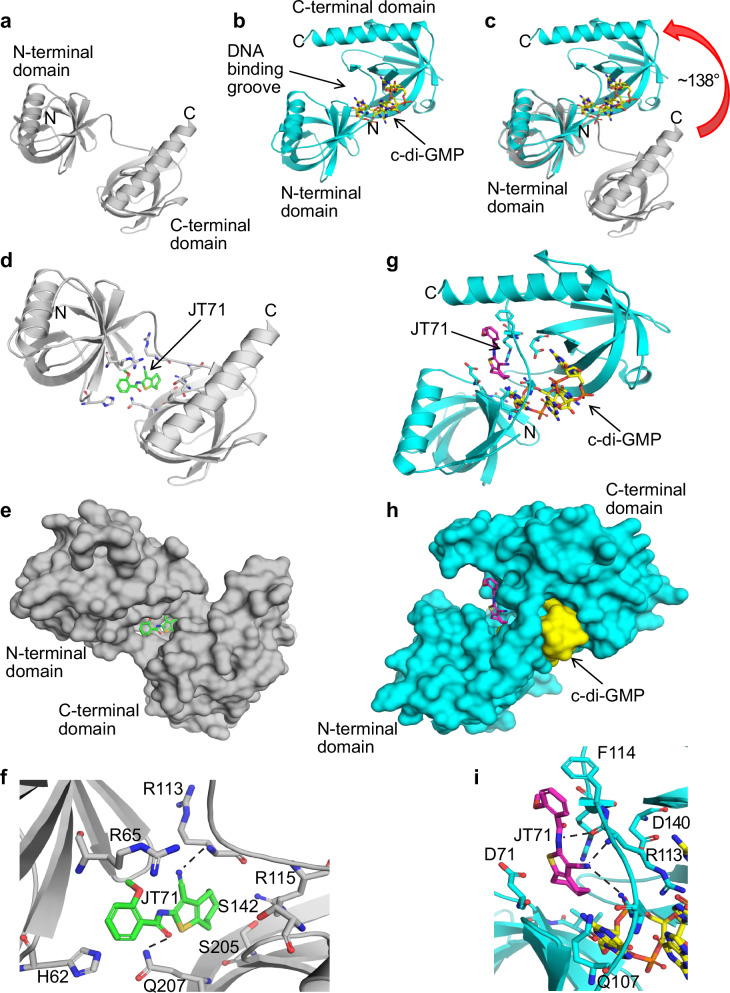
Potential inhibitory mechanisms of JT71. The *apo*- and *holo*-MrkH structures are shown in (**a**, **b**), respectively (PDB ID: 5KEC and 5KGO^55^). The N- and C-terminal PilZ domains are indicated. **b** The two intercalated molecules of c-di-GMP bound to the *holo*-MrkH are depicted as yellow sticks, and the putative DNA binding groove is labelled. **c** Overlay of the N-terminal PilZ-like domain of *apo*- and *holo*-MrkH illustrating the large domain movement (~138°) that occurs upon binding the two molecules of c-di-GMP to the C-terminal PilZ domain. The root mean square deviation for the overlay of the *apo* and *holo* N-terminal PilZ-like domains (Cα atoms) is 0.4 Å. **d**–**f** In *apo*-MrkH, JT71 (green sticks) can bind to a pocket located between the N- and C-terminal PilZ domains. **g**–**i** In *holo*-MrkH, the DNA binding groove is the likely site of interaction of JT71 (magenta sticks). The two molecules of c-di-GMP form part of the putative DNA-binding groove and the JT71 binding pocket. **e**, **h** Show the same view as (**d**, **g**), respectively, with MrkH and c-di-GMP depicted as molecular surfaces. **f**, **i** Close-up view of the two proposed JT71 binding pockets shown in (**d**, **g**), nearby residues are shown as sticks. Polar interactions are depicted as black dashed lines. **f** H62 can interact with the phenyl ring of JT71 via a π-π interaction, whereas in **i** F114 makes this interaction with JT71.

The structure of *K. pneumoniae* AJ218 MrkH is comprised of two distinct domains connected by a six-residue flexible linker (Fig. 5a)^55^. The C-terminal domain adopts a traditional PilZ domain fold, whereas the fold of the N-terminal domain has been described as PilZ-like. Binding two intercalated molecules of c-di-GMP to the C-terminal PilZ domain of *apo-*MrkH (i.e., inactive MrkH) induces a domain rotation, which then enables the N-terminal domain to also interact with the c-di-GMP molecules (Fig. 5a–c)^55^. Directly opposite the two c-di-GMP molecules is a groove that runs across the central section of *holo*-MrkH (i.e., active MrkH, Fig. 5b). The groove is electrostatically positive (lined by several basic lysine and arginine residues), typical of a DNA-binding groove^55^.

JT71 could potentially inhibit MrkH activity by two distinct mechanisms. The first mechanism involves JT71 binding to the *apo* form of MrkH, thereby stabilising this conformation. The putative binding site of JT71 in *apo*-MrkH is a pocket located between the N- and C-terminal PilZ domains (Fig. 5d, e), which overlaps with the binding site of the two intercalated molecules of c-di-GMP. Residues forming this proposed JT71 binding site are F32, N34, H62, K63, R65, N78, P112, R113, F114, R115, S142, D143, G144, K184, N185, S205, C206 and Q207. Modelling revealed these residues can interact with JT71 via polar interactions, π-π interactions, and Van der Waals contacts (Fig. 5f). Once bound, JT71 could prevent or impede c-di-GMP from binding, and thereby block the formation of *holo*-MrkH and subsequent DNA binding.

An alternative inhibitory mechanism may involve JT71 binding to the proposed DNA-binding groove of *holo*-MrkH (Fig. 5g), preventing DNA from accessing the site and thereby inhibiting MrkH. The two molecules of c-di-GMP also form part of the putative DNA-binding groove. The electropositive DNA binding groove provides a snug binding pocket for JT71 (Fig. 5g, h), where it is surrounded by residues K25, R26, E28, H69, D71, Q107, R109, R110, P112, R113, F114, R115, R117, and D140. These residues, along with nearby atoms of c-di-GMP, can interact with JT71 via polar interactions, π-π interactions, and Van der Waals contacts (Fig. 5i).

### JT71 inhibits biofilm formation independently of c-di-GMP abundance

**Fig. 6:**
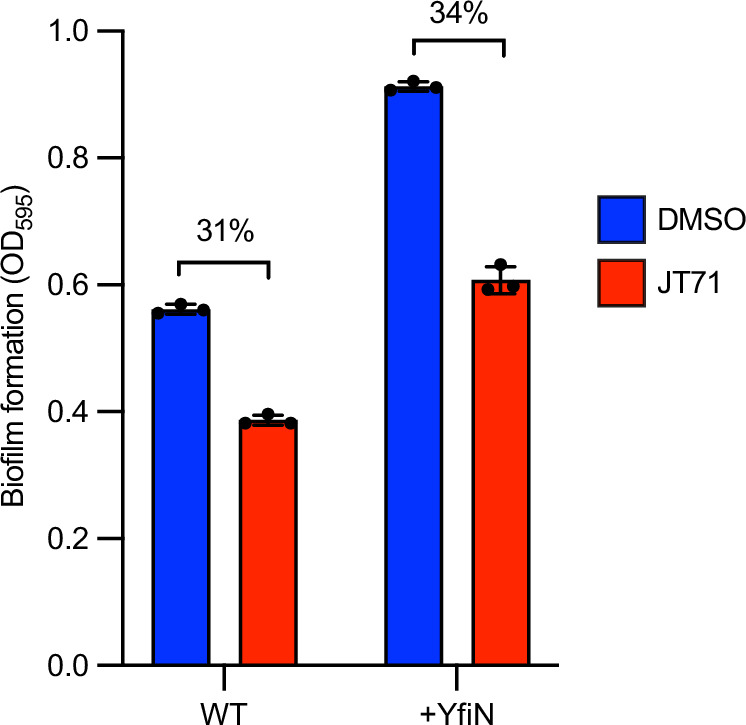
Biofilm formation of *K. pneumoniae* AJ218. Biofilm biomass (OD_600_) was measured after 24 hours for AJ218 wild-type (WT) and AJ218 containing pACYC184-*yfiRNB*, which expresses the diguanylate cyclase YfiN to elevate intracellular c-di-GMP and promote biofilm formation. Cultures were treated with 50 μM JT71 or 1% DMSO. The percentage reductions caused by JT71 treatment are shown. Data represent the mean ± SD of three biological replicates, each averaged from five technical replicates.

To further explore the mechanism by which JT71 inhibits MrkH-dependent biofilm formation, we compared its activity in two *K. pneumoniae* strains, AJ218 wild-type and AJ218 expressing the diguanylate cyclase YfiN (expressed from pACYC184-*yfiRNB*), which elevates intracellular c-di-GMP and enhances biofilm formation^56^. If JT71 acted by competing with c-di-GMP at MrkH (as illustrated in Fig. 5d–f), its inhibitory effect would be expected to diminish in the +YfiN strain, where excess c-di-GMP could outcompete the compound. Figure 6 shows similar magnitudes of biofilm inhibition, which suggests that JT71 may not primarily compete with c-di-GMP binding, but instead interferes with the DNA-binding activity of MrkH (as illustrated in Fig. 5g–i).

## Discussion

Indwelling medical devices such as implants, catheters, and endotracheal tubes are frequently used in medical practice. With prolonged use, these devices can be colonised with microbial biofilms, commonly leading to chronic infections that are difficult to treat. *K. pneumoniae* is an opportunistic pathogen that is often associated with nosocomial infections amongst patients with indwelling devices^6,57,58^. A study on device-related infections showed that *K. pneumoniae* was the major contributor to biofilm-based catheter-related bloodstream infections and catheter-associated urinary tract infections^6^, and KpSC are commonly isolated from reused catheters in patients with spinal injury^59^.

Biofilm-associated cells have considerably reduced susceptibility to antimicrobial agents and disinfectants. Factors that contribute to this phenomenon include decreased antimicrobial diffusion through the biofilm matrix and the reduced growth rate of bacterial cells within the biofilm^60–62^. The biofilm environment also provides a supportive niche for enhanced conjugative plasmid transfer, further increasing the likelihood of antimicrobial resistance gene transmission between bacteria in multi-species biofilms^63,64^. These observations highlight the need to develop a more complete understanding of biofilm development and new approaches to prevent and treat bacterial biofilm-related infectious diseases.

The coating of catheters with antimicrobial substances has had some impact on biofilm formation^65^, and we hypothesised that a compound that specifically inhibits a key determinant of the biofilm process might have better utility by preventing initial adhesion of bacterial cells. Both initial and mature biofilm formation by *K. pneumoniae* has been shown to require the expression of type 3 fimbriae^33–35^. The MrkA filament of type 3 fimbriae mediates adherence to abiotic surfaces, while MrkD mediates attachment to specific host cell receptors on damaged tissue surfaces and/or catheters coated in situ with host-derived extracellular matrix proteins^33,37,38,66^. Studies have demonstrated that defined mutants of *K. pneumoniae* that lack either MrkA or MrkD can no longer effectively form biofilms on uncoated or human collagen-coated surfaces, respectively^37,39^. The type 3 fimbriae operon forms part of the *K. pneumoniae* core genome and was conserved in over 300 global isolates analysed^67^. Blocking type 3 fimbriae production represents a clear strategy to prevent *K. pneumoniae* adhesion to surfaces and subsequent infection in susceptible hosts.

The MrkH transcriptional regulator activates the expression of type 3 fimbriae^39,40^. In previous studies, we showed that MrkH becomes activated by c-di-GMP and initiates transcription at both the *mrkA* and *mrkH* promoters^39,42,43^. On this basis, we developed a high-throughput phenotypic screening assay that employed a transcriptional reporter to identify small molecules that block MrkH function. The main benefit of using small molecule inhibitors that specifically target regulatory pathways contributing to virulence (e.g., adhesion) is that bacterial growth is unlikely to be inhibited, thus reducing selective pressure for the development of resistance. A small molecule screening approach has been validated by other groups seeking biofilm inhibitors of *Vibrio cholerae*^68^, *Porphyromonas gingivalis*^69^, *Candida albicans*^70^, *Listeria monocytogenes*^71^, *Streptococcus mutans*^72^, and *Pseudomonas aeruginosa*^73^.

In this study, we identified a compound, JT71, that inhibited MrkH activity and subsequently reduced transcription of the type 3 fimbriae *mrk* operon. Of the 13,440 compounds screened, JT71 was the only molecule that consistently and selectively reduced *mrkA-lacZ* reporter expression. This low hit frequency suggests that pharmacological inhibition of MrkH-dependent *mrk* expression may be challenging, or that such inhibitors are rare within the limited chemical space represented in the screened library. We also showed that *mrkH* gene expression itself was down-regulated in the presence of JT71. Previously, we reported that MrkH also auto-regulates *mrkH* gene expression^43^. The two inhibitory targets suggest that the mode of transcription inhibition by JT71 of the *mrkHI* and *mrkABCDF* operons is similar. The observed transcriptional inhibition of *lacZ* reporter fusions by JT71 supports the conclusion that the compound enters the bacterial cytosol. Since β-galactosidase activity is a cytoplasmic process, any reduction in reporter output implies intracellular interference with the transcriptional machinery. This is consistent across both the *E. coli* screen and follow-up assays in *K. pneumoniae*, and occurred in the absence of growth inhibition, indicating that the effect is not due to general toxicity.

We also demonstrated that *K. pneumoniae* can form robust biofilms on a variety of medically relevant materials, and that MrkH is critical for this process^43^. By selecting MrkH as a target to inhibit type 3 fimbriae synthesis, we identified a compound, JT71, that inhibited biofilm formation on biotic (human collagen) and abiotic (polystyrene) surfaces commonly encountered by *K. pneumoniae*. We also showed that JT71 can inhibit biofilms of other clinical *K. pneumoniae* isolates in our collection.

Although JT71 shows promising anti-biofilm activity against *K. pneumoniae* in vitro, a key limitation of many antivirulence strategies is the challenge of demonstrating their effectiveness in vivo. While type 3 fimbriae are well-established mediators of adhesion to abiotic surfaces and extracellular matrix components, their contribution to virulence in standard murine models remains poorly defined. Further evaluation in catheter- or device-associated infection models may provide more appropriate systems to assess the in vivo utility of JT71. Moreover, to enhance the therapeutic potential of JT71, additional in vitro studies are warranted. We examined the importance of the ortho-methoxy substituent on biofilm formation using a small set of JT71 analogues, however, none of the eight analogues were as active as the parent compound. Optimisation of the in vitro activity and physicochemical properties of JT71 through extensive structure-activity relationship (SAR) analysis would be an important step before progressing to animal models and assessing bioavailability, efficacy, and toxicity.

Early-stage biofilm formation was effectively inhibited by JT71; however, additional testing showed that it does not disrupt pre-formed biofilms, even with prolonged exposure. This indicates that its activity is confined to blocking MrkH-dependent type 3 fimbriae during the initial stages of adhesion rather than dismantling mature biofilms dominated by matrix components. Molecules such as JT71 may therefore be best suited as prophylactic anti-biofilm agents, for example, through incorporation into catheter or implant coatings to prevent initial bacterial colonisation.

Like *K. pneumoniae*, *C. koseri* forms biofilms through expressing type 3 fimbriae, with the *mrkHI* and *mrkABCDF* gene clusters well conserved^51^. We speculated that the MrkH homologue of *C. koseri* has a similar function and mechanism of action to that of *K. pneumoniae* MrkH. In support of this, we observed a significant reduction in biofilm formation by *C. koseri* when treated with JT71, suggesting that *Citrobacter* MrkH functions as a transcriptional activator of the *C. koseri mrk* genes.

A variety of compounds have been shown to reduce *K. pneumoniae* biofilm formation. Some compounds have been proposed to target iron uptake mechanisms^32,74^ or stress response regulators^75^. However, the mode of action for the majority of biofilm inhibitors reported is currently unknown. Type 3 fimbriae are synthesised by the chaperone-usher pathway of protein translocation and belong to the same family as the well-characterised P-pili (*pap*) and type 1 fimbriae (*fim*) systems. Various chemicals have been shown to inhibit *E. coli* attachment to intestinal cells by blocking the FimH adhesin of type 1 fimbriae^76,77^, or to inhibit the assembly of type 1 or AAF/II fimbriae post-translationally^78,79^. A general ‘pilicide’ has been developed which inhibits piliation by *E. coli* type 1, P and S pili^80^, and quorum sensing inhibition has also proved useful in blocking pili production^81,82^. To our knowledge, JT71 is the first reported chemical inhibitor of type 3 fimbriae transcription and biosynthesis, and we present two models illustrating putative binding sites of JT71 on *apo* or *holo*-MrkH.

One of the most important signalling molecules produced by bacteria is c-di-GMP, because it regulates the lifestyle transition between planktonic and biofilm states^83–87^. The ‘PilZ-domain’ proteins are the most common c-di-GMP-binding effector proteins identified so far. These proteins regulate diverse processes, including fimbriae production, flagellar activity, exopolysaccharide synthesis, and gene regulation^83^. Although functionally diverse, all PilZ proteins share a highly conserved sequence and structural homology in their c-di-GMP binding site. Because c-di-GMP signalling is highly conserved and unique in bacteria, and has a central role in controlling biofilm formation, targeting this system provides an attractive approach to inhibit bacterial adherence. Compounds have been identified that interfere with c-di-GMP metabolism by binding to diguanylate cyclase^31,88^ and phosphodiesterase^73^ proteins to reduce c-di-GMP synthesis, as well as binding to the PilZ-domain protein Alg44 from *P. aeruginosa* to reduce alginate production^89^.

We have identified a specific inhibitor, JT71, which targets MrkH-mediated activation of transcription at two genetic loci, and results in reduced type 3 fimbriae synthesis and biofilm formation by *K. pneumoniae*. This inhibition of biofilm formation extended over a wide range of clinically relevant isolates. Given the widespread conservation of type 3 fimbriae amongst *K. pneumoniae*, and the high prevalence of c-di-GMP-associated factors produced by the majority of bacterial species, this proof-of-principle study provides a foundation for further design and development of agents that reduce the risk of biofilm-associated infections without exacerbating the problem of more generalised antibiotic resistance.

## Methods

### Bacterial strains, plasmids, and growth conditions

The bacterial strains and plasmids used in this study are listed in Supplementary Table S1. *K. pneumoniae* AJ218 is a human urinary tract infection isolate^90^. Unless otherwise stated, all bacterial strains were cultured at 37 °C, with shaking, in Luria Bertani (LB) broth overnight, followed by dilution in M63B1-GCAA minimal media (M63; containing 1% glycerol and 0.3% casamino acids). When necessary, the growth medium was supplemented with chloramphenicol (30 µg/mL) or trimethoprim (200 µg/mL).

### Small molecule screening assay

Overnight cultures of the test strain *E. coli* MC4100 (containing pMU*mrkA*-*lacZ* and pACYC184-*mrkH*)^39^ and the control strain *E. coli* MC4100 (containing pMU*aap-lacZ* and pACYC184-*aggR*) were diluted 1:100 in LB broth containing chloramphenicol and trimethoprim and then dispensed in 100 μL aliquots into separate 96-well microtiter trays. Compounds (5 µL, 2 mM) from the ChemBridge Microformats library (ChemBridge, San Diego, USA) were added to wells (100 μM final concentration), and trays were incubated at 37 °C for 10 h. The compound library was dissolved in 100% DMSO, resulting in a final DMSO concentration of 5%. An 8 μL aliquot of lysozyme (6 mg/mL) was added to wells and incubated for 20 min to lyse the bacterial cells. A 25 μL aliquot of Beta-Glo solution (Promega, Madison, USA) was then added to wells to convert the β-galactosidase released from cells into a luminescence signal. The level of luminescence from each well was measured at 0 s, 71 s, and 142 s with a FLUOStar Omega plate reader (BMG Labtech, Offenburg, Germany). Compounds that inhibited luminescence by >50% (measured as Relative Luminescence Units (RLU) compared to a representative non-inhibitory compound on the same plate), without reducing the RLU of the control strain by >30% relative to the same reference, at either of the two time-points, were identified as promising candidates and investigated further. DMSO-only controls showed no inhibition of growth or β-galactosidase activity, indicating *E. coli* was tolerant under the assay conditions. For subsequent validation experiments in *K. pneumoniae*, DMSO was used at 1% to minimise solvent-related effects.

### Beta-galactosidase assay

Overnight cultures were diluted 1:100 with M63 media containing the appropriate antibiotics and grown statically in the presence or absence of the ChemBridge compound at 37 °C for 6 h (OD_600_ ~ 0.8), after which the β-galactosidase activity was determined as described elsewhere^39^. The data shown are the results of three independent assays.

### RNA extraction

*K. pneumoniae* AJ218 was grown statically overnight at 37 °C in M63 supplemented with either 50 µM JT71 or 1% DMSO. Bacteria were diluted 1:100 in the same media and supplements, and grown in 96-well microtiter trays statically at 37 °C for 6 h (OD_600_ of ~0.9). A 5 mL volume of culture was incubated with 10 mL of RNAProtect solution (Qiagen, Hilden, Germany) at room temperature. The cells were pelleted, and RNA was extracted using a FastRNA Pro Blue Kit (Q-Biogene). Residual DNA was removed using an RNase-free DNase set (Qiagen) prior to RNA purification with a RNeasy MiniElute Cleanup Kit (Qiagen). RNA quality was validated with an Agilent 2100 Bioanalyzer (G2938C). All samples had an RNA Integrity Number (RIN) greater than 8.1.

### Quantitative real-time PCR

The extracted cellular RNA from *K. pneumoniae* AJ218 (1 µg) was used as template to synthesise cDNA with Superscript II Reverse Transcriptase (Life Technologies, USA) with random hexamers in accordance with the manufacturer’s instructions. The absence of residual genomic DNA was verified by a conventional PCR reaction. qRT-PCR was performed using a 20 μL reaction mixture containing the following: 10 μL of 2× SsoFast Evagreen Supermix (Bio-Rad Laboratories, California, USA), 1 μM of primer pair qMrkA-for (AGGATGACGTAAGCAAACTGG) and qMrkA-rev (CGGTAGCGTTGTCAGTAGACAG) or qrpoDFor (TAAGGAGCAAGGCTATCTGACC) and qrpoDRev (ACCTGAATACCCATGTCGTTG), and 5 μL of 1:10 diluted of newly synthesised first strand cDNA. Each reaction was performed in triplicate. Amplification of target and housekeeping gene transcripts was performed with a CFX96 RT-PCR/C1000 thermal cycler (Bio-Rad Laboratories) with the following programme: 95 °C for 30 s, followed by 40 cycles of 95 °C for 5 s and 55 °C for 20 s. CFX Manager version 2.0 (Bio-Rad) software was used to perform data analysis. The gene of interest (*mrkA* or *mrkH*) was normalised against the housekeeping gene, *rpoD,* and the expression ratios between untreated (1% DMSO) and treated (50 µM JT71) samples were determined.

### Western blot

Overnight cultures were diluted 1:100 with M63 media with appropriate antibiotics in 96-well microtiter plates. Following 6 h incubation at 37 °C, cells were harvested, and the OD_600_ of the whole-cell lysates was standardised. Samples were separated by SDS-PAGE and transferred to Hybond-C Extra nitrocellulose (Amersham Bioscience, Amersham, UK) using a Transblot SD Electrophoretic Transfer Cell (Bio-Rad Laboratories) at 12 V for 30 min. The membrane was blocked for 2 h at 4 °C in 5% skim milk in PBS and then washed 3 × 10 min in 0.05% PBST, followed by 3 × 10 min in distilled water. The primary MrkA antibody (anti-rabbit) was diluted 1:1000 in PBS containing 1% casein and 0.05% thiomersal and incubated with the membrane at 4 °C overnight. Unbound antibody was removed by washing the membrane in 0.05% PBST for 3 × 15 min, followed by 3 × 10 min in distilled water. Anti-rabbit secondary antibody (goat anti-rabbit IgG) conjugated to horseradish peroxidase (Invitrogen) was diluted 1:3000 with 1% skim milk in PBS and incubated with the membrane for 2 h. The membrane was subsequently developed with TMB Membrane Peroxidase Substrate (KPL, Gaithersburg, USA).

### Hemagglutination assay

Type 3 fimbriae production by *K. pneumoniae* was detected by mannose-resistant hemagglutination assays as described previously^39^. Briefly, tannic acid-treated sheep erythrocytes were mixed with equal volumes of a series of 2-fold dilutions of bacterial suspension treated with 50 μM of JT71 or 1% DMSO. The minimum bacterial density (cfu/mL) required to agglutinate erythrocytes was determined. Two independent experiments were performed, yielding a total of eight samples. The data shown are representative of one experiment (five samples).

### Static biofilm assay

Nunc 96-well non-treated polystyrene microtiter plates (Thermo Scientific, Massachusetts, USA) that were uncoated or coated with type IV human collagen (Sigma-Aldrich; C7526) were used for biofilm assays. The type IV collagen-binding assay was performed as described previously^91,92^ with minor modifications. Briefly, wells were coated with type IV collagen (5 μg/mL) in PBS for 18 h at 4 °C, followed by a blocking step with 1% BSA for 2 h at room temperature. The wells were then washed three times with 0.05% PBST and air-dried. Material surfaces were incubated with ~ 10^7^ cfu/mL of bacteria in M63 media supplemented with JT71 (10–100 µM) or its corresponding DMSO concentration (0.2–2%). Following 6 h of static incubation at 37 °C, the surfaces were washed twice with distilled water. Adherent biofilms were stained with 0.1% crystal violet solution (Sigma-Aldrich, Missouri, USA) for 15 min, solubilised with 33% acetic acid, and absorbance readings were taken at OD_600_. Data for each strain represents mean values taken from at least five replicate wells performed in at least three independent experiments.

### Transmission electron microscopy

Overnight cultures of wild-type *K. pneumoniae* AJ218 were diluted 1:100 with M63 media supplemented with 50 μM of JT71 or 1% DMSO. Following 6 h of static incubation at 37 °C, the cultures were pelleted and resuspended with 100 μL of PBS. 15 μL of the suspension was placed on formvar/carbon-coated EM grids. The grids were blocked with blocking solution (1% skim milk, 1% BSA in TBS) for 20 min at room temperature and transferred directly to primary MrkA antibody (1:100) for 1 h incubation. The grids were subsequently washed with washing buffer (TBS with 0.2% BSA) and incubated with gold conjugate (1:100 in TBS + 0.2% BSA), after which the samples were negatively stained with 1.5% ammonium molybdate for 30 s and placed under a Phillips CM120 transmission electron microscope at 120 kV for visualisation.

### Synthesis of *N*-(3-cyano-5,6,7,8-tetrahydro-4H-cyclohepta[b]thien-2-yl]-2-hydroxybenzamide (JT71-OH)

To a solution of *N*-(3-cyano-5,6,7,8-tetrahydro-4H-cyclohepta[b]thien-2-yl]-2-methoxybenzamide (JT71) (1.0 mM, 328 mg) in anhydrous dichloromethane (DCM) (20 mL) under argon at −78 °C was added boron tribromide (1.0 M in DCM, 2.0 mL) dropwise. The reaction was allowed to warm to room temperature and stirred for a further 22 h. Water (20 mL) and diethyl ether (20 mL) were added, and the phases were separated. The aqueous phase was extracted with diethyl ether (3 × 20 mL), and the combined organic phases were washed with water and brine, dried (MgSO_4_), and evaporated to give a yellow-brown powder. Flash column chromatography (silica gel, EtOAc-hexane, 4:1) gave *N*-(3-cyano-5,6,7,8-tetrahydro-4H-cyclohepta[b]thien-2-yl]-2-hydroxybenzamide (JT71-OH) as a white powder (314 mg, 100%).

### Eukaryotic cell toxicity assay

An LDH Cytotoxicity Detection Kit (Takara Bio Inc.; Japan) was used to determine the release of lactate dehydrogenase (LDH), an indicator of cell death. The assay was performed according to the manufacturer’s instructions. Briefly, HeLa cells in DMEM (10% FCS, v/v) were seeded at ~0.8 × 10^4^ cells/well. After overnight incubation at 37 °C with 5% CO_2_, the cells were washed once with PBS to remove LDH released during overnight incubation. The cells were then supplemented with DMEM (1% FCS, v/v) only, or media containing JT71 (50 μM) or its corresponding DMSO concentration. Controls included cells incubated with DMEM (1% FCS, v/v) supplemented with 1% Triton X-100 or DMEM (1% FCS, v/v) in empty wells. After incubation for 6 h, the microtiter plates were centrifuged at 250 × *g* for 10 min, and 100 μL/well supernatant was transferred to new wells. 100 μL of the substrate mix was added to the wells and incubated up to 15 min. Absorbance (at 490 nm) from each well was measured using a FLUOStar Omega plate reader. The percentage of cell death (cytotoxicity) was determined by using the formula $\frac{({experimental\; value}-{background\; release})}{({maximum\; release}-{background\; release})}$ × 100%.

### Modelling the JT71–MrkH complex

There are four MrkH crystal structures in the Protein Data Bank (PDB) for *K. pneumoniae* K54 strain AJ218 (UniProt ID: G3FT00): two are *apo* (inactive) structures (PDB IDs: 5KEC and 5KED^55^) and two are *holo* (active) structures where MrkH is in complex with two intercalated molecules of c-di-GMP (PDB ID: 5EJL^93^ and 5KGO^55^). In the *apo*-MrkH structures, there are two MrkH molecules in the 5KEC asymmetric unit (the smallest repeating object that generates the unit cell of the crystal) and four such molecules in 5KED, giving a total of six separate molecules to examine. The six molecules of *apo*-MrkH were aligned via the Cα atoms (root mean square deviation <0.8 Å); the conformations are almost identical, therefore the A chain of 5KEC was selected for the compound JT71 in silico docking studies. In the asymmetric unit of the unit cell of the *holo*-MrkH complexes, there are two molecules of MrkH in 5KGO and one molecule in 5EJL. The three molecules of *holo*-MrkH were aligned via the Cα atoms (root mean square deviation <1.7 Å); there are small conformational differences at the extremities of the N- and C-terminal MrkH PilZ domains, but these are well away from the c-di-GMP binding pocket and the putative DNA binding groove. The three molecules of *holo*-MrkH are very similar around the c-di-GMP binding pocket and the putative DNA binding groove; therefore, the A chain of 5KGO was selected for JT71 in silico docking studies.

JT71 was docked into the two most distinct putative binding pockets, one located between the N- and C-terminal PilZ domains of *apo*-MrkH, and the second was the putative DNA binding groove of *holo*-MrkH. Flexible protein and compound docking was performed using Surflex-Dock v2.7 (within SYBYL-X 2.1.1, Certara, L.P., Princeton, NJ, USA); the protocol was generated using the automatic method, a 0.5 threshold, and a bloat value of 3 for *apo*-MrkH and 5 for *holo*-MrkH. GeomX docking mode was used, with protein flexibility enabled and all other parameters set to default values. The docked poses of JT71 were ranked using the C Score scoring function, and the top 30 ranked poses in each MrkH structure (i.e., the apo and holo structures) were retained for analysis.

All JT71-MrkH docking figures were created using The PyMOL Molecular Graphics System, Version 2.1.1 (Schrödinger LLC, Cambridge, MA).

### Physicochemical properties of JT71

Molecular weight (g/mol), partition coefficient (clogP), topological polar surface area (tPSA in Å^2^), and the number of hydrogen bond donors and acceptors were calculated for JT71 using OSIRIS DataWarrior version 5.2.1^94^.

## Supplementary information

Supplementary information

## Data Availability

Data is provided within the manuscript or supplementary information files.
