# Supplementary material for: Chemical inhibition of MrkH-dependent activation of type 3 fimbriae synthesis and biofilm formation by Klebsiella pneumoniae
Source: NPJ Biofilms Microbiomes. 2025 Nov 20;11:212. doi: 10.1038/s41522-025-00834-3 (PMC12635112; doi:10.1038/s41522-025-00834-3)
Supplement: Supplementary file 1 — Supplementary information [file 41522_2025_834_MOESM1_ESM.pdf]

Supplementary Information

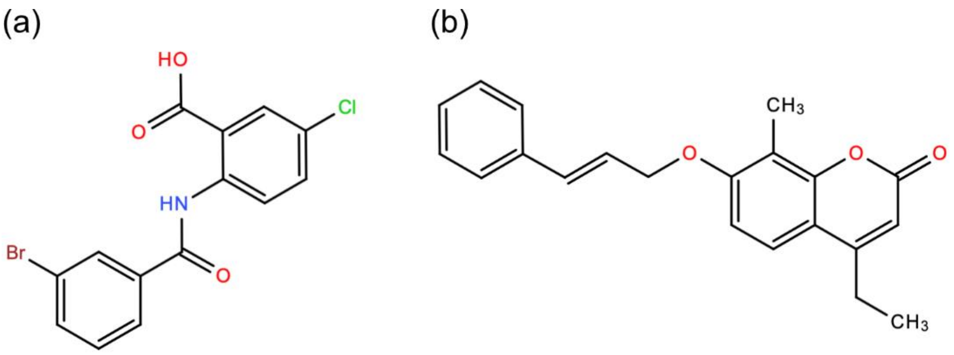

Fig. S1. Chemical structures of representative screen compounds. (a) B371: 2-[(3-bromobenzoyl)amino]-5-chlorobenzoic acid. **(b)** G771: 4-ethyl-8-methyl-7-[(3-phenyl-2-propen-1-yl)oxy]-2H-chromen-2-one.

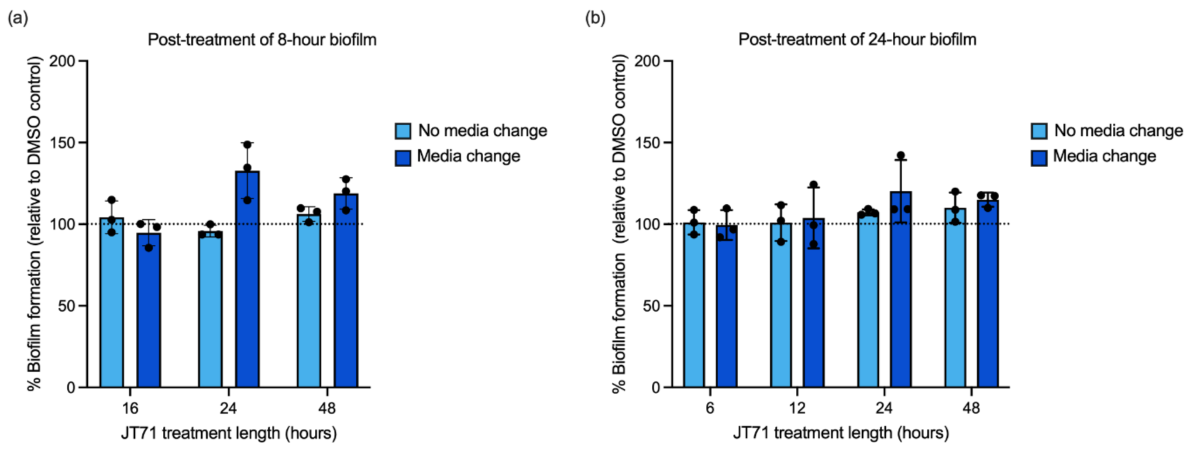

Fig. S2. Effect of JT71 treatment on pre-formed biofilms. *K. pneumoniae* AJ218 biofilms were allowed to form for **(a)** 8 hours or **(b)** 24 hours prior to treatment with 50 µM JT71 or 1% DMSO. Biofilms were incubated for up to 48 hours post-treatment, with drug added directly to the existing medium ('no media change') or after replacement with fresh drug-containing medium ('media change'). Biofilm biomass is expressed as a percentage of the matched DMSO control (same timepoint and media condition). Bars represent mean ± SD of three biological replicates (each the mean of five technical replicates); points show biological replicate mean. No significant differences from controls were observed (Wilcoxon signed-rank test vs 100%, $P > 0.05$).

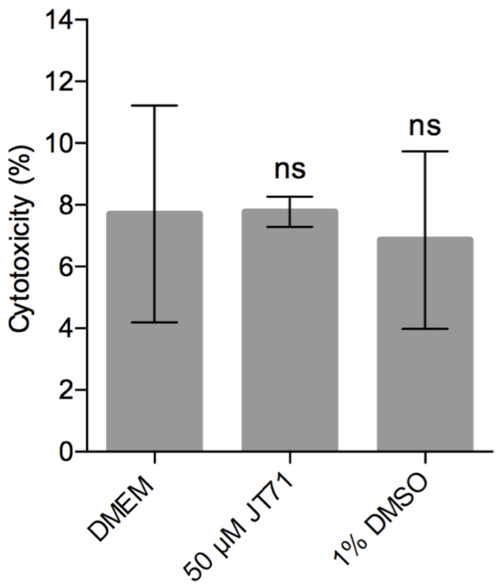

Fig. S3. JT71 is non-toxic to mammalian cells. HeLa cells were incubated with 50 µM JT71 or 1% DMSO for 6 hours. Cell supernatants were then quantified for lactate dehydrogenase (LDH) release. The percentage of LDH release from treated cells was compared with untreated cells using one-way ANOVA with Bonferroni post-test, ns, not significant ($P > 0.05$). Data are mean ± S.D of three independent assays.

Supplementary Table S1: Strains and plasmids used in this study

**Strain or plasmid**	**Relevant phenotypes and genotypes**	**Source or reference**
**Strains**		
K. pneumoniae AJ218	Human clinical isolate, serotype K54	1
K. pneumoniae AJ97	Human clinical isolate, serotype K2	1
K. pneumoniae AJ94	Human clinical isolate, serotype K2	1
K. pneumoniae Δ*mrkA*	AJ218 *mrkA* deletion mutant	2
K. pneumoniae MGH 78578	Human clinical isolate	Salmonella Genetic Stock Center
E. coli MC4100	Δ(*argF-lac*)*U169*, *rpsL150*, *relA*, *araD139*, *fib5301*, *deoC1*, *ptsF25*	3
C. koseri BAA-895	Human clinical isolate	American Type Culture Collection
P. aeruginosa PAO1	Human clinical isolate	4
**Plasmids**		
pMU2385	*galK'-lac'Z*, IncW, single-copy-no. transcriptional-fusion vector; Tp^R	5
pACYC184	Medium-copy-no. cloning vector, p15A ori; Tet^R Chl^R	6
pACYC177	Medium-copy-no. cloning vector, p15A ori; Ap^R Kn^R	6
pACYC184-*mrkH*	*K. pneumoniae* AJ218 *mrkH* cloned into pACYC184; Chl^R	2
pACY184-*yfiRNB*	*K. pneumoniae* AJ218 *yfiRNB* cloned into pACYC184; Chl^R	2
pACYC184-*aggR*	Enteroaggregative *E. coli aggR* cloned into pACYC184; Chl^R	This study
pACYC177-*tyrR*	*E. coli* K-12 *tyrR* cloned into pACYC177; Kn^R	7
pMU*mrkA-lacZ*	*mrkA-lacZ* transcriptional fusion vector (pMU2385) from *mrkA* promoter nucleotides -91 to +313 relative to the *mrkA* transcriptional start site; Tp^R	2
pMU*aaP-lacZ*	*aaP-lacZ* transcriptional fusion vector (pMU2385) from *aaP* promoter nucleotides -314 to +83 relative to the *aaP* translational start site; Tp^R	This study
pMU*mtr-lacZ*	*mtr* promoter region in pMU2385, Tp^R	7

Abbreviations: Tet: tetracycline; Chl: chloramphenicol; Ap: ampicillin; Kn: kanamycin; Tp: trimethoprim.

References

1. Jenney, A. W. *et al.* Seroepidemiology of *Klebsiella pneumoniae* in an Australian Tertiary Hospital and its implications for vaccine development. *J. Clin. Microbiol.* **44**, 102-107 (2006).
2. Wilksch, J. J. *et al.* MrkH, a novel c-di-GMP-dependent transcriptional activator, controls *Klebsiella pneumoniae* biofilm formation by regulating type 3 fimbriae expression. *PLoS Pathog.* **7**, e1002204 (2011).
3. Casabadan, M. J. Transposition and fusion of the *lac* genes to selected promoters in *Escherichia coli* using bacteriophage lambda and Mu. *J. Mol. Biol.* 541-555 (1976).
4. Holloway, B. W. Genetic recombination in Pseudomonas aeruginosa. J Gen Microbiol. **13**, 572-581 (1955).
5. Yang, J., Tauschek, M., Strugnell, R. & Robins-Browne, R. M. The H-NS protein represses transcription of the *eltAB* operon, which encodes heat-labile enterotoxin in enterotoxigenic *Escherichia coli*, by binding to regions downstream of the promoter. *Microbiology* **151**, 1199-1208 (2005).
6. Chang, A. C. & Cohen, S. N. Construction and characterization of amplifiable multicopy DNA cloning vehicles derived from the P15A cryptic miniplasmid. *J. Bacteriol.* **134**, 1141-1156 (1978).
7. Yang, J. *et al.* Disarming bacterial virulence through chemical inhibition of the DNA binding domain of an AraC-like transcriptional activator protein. *J. Biol. Chem.* **288**, 31115-31126 (2013).
